# Diffuse Noxious Inhibitory Controls in Chronic Pain States: Insights from Pre-Clinical Studies

**DOI:** 10.3390/ijms26010402

**Published:** 2025-01-05

**Authors:** Raquel Pereira-Silva, Fani L. Neto, Isabel Martins

**Affiliations:** 1 Instituto de Investigação e Inovação em Saúde da Universidade do Porto–i3S, R. Alfredo Allen 208, 4200-135 Porto, Portugal; raquel.silva.farm.86@gmail.com; 2 Instituto de Biologia Molecular e Celular (IMBC), Universidade do Porto, R. Alfredo Allen 208, 4200-135 Porto, Portugal; 3 Departamento de Biomedicina, Unidade de Biologia Experimental, Faculdade de Medicina, Universidade do Porto, Al. Prof Hernâni Monteiro, 4200-319 Porto, Portugal

**Keywords:** conditioned pain modulation, descending pain modulation, noradrenaline, serotonin, opioids, brainstem, chronic pain

## Abstract

Diffuse noxious inhibitory control (DNIC), also known as conditioned pain modulation (CPM) in humans, is a paradigm wherein the heterotopic application of a noxious stimulus results in the attenuation of another spatially distant noxious input. The pre-clinical and clinical studies show the involvement of several neurochemical systems in DNIC/CPM and point to a major contribution of the noradrenergic, serotonergic, and opioidergic systems. Here, we thoroughly review the latest data on the monoaminergic and opioidergic studies, focusing particularly on pre-clinical models of chronic pain. We also conduct an in-depth analysis of these systems by integrating the available data with the descending pain modulatory circuits and the neurochemical systems therein to bring light to the mechanisms involved in the regulation of DNIC. The most recent data suggest that DNIC may have a dual outcome encompassing not only analgesic effects but also hyperalgesic effects. This duality might be explained by the underlying circuitry and the receptor subtypes involved therein. Acknowledging this duality might contribute to validating the prognostic nature of the paradigm. Additionally, DNIC/CPM may serve as a robust paradigm with predictive value for guiding pain treatment through more effective targeting of descending pain modulation.

## 1. Introduction

The concept of diffuse noxious inhibitory control (DNIC) first appeared in the field of pain research through the studies of Le Bars et al. [1,2,3,4,5]. DNIC is known as a paradigm of endogenous analgesia that is driven by the “pain inhibits pain” principle. This experience occurs when an additional painful stimulus in one part of the body suppresses the initial spatially distant experienced pain [6,7]. In the literature, this phenomenon is also mentioned as counter-irritation or heterotopic noxious counter-stimulation [6]. DNIC involves supraspinal circuitries that encompass several cortical areas identified in human imaging studies [8], along with brainstem regions involved in descending modulation [9].

In humans, DNIC is commonly known as conditioned pain modulation (CPM), which is a psychophysical paradigm based on quantitative sensory tests to assess the functionality of endogenous pain inhibition [10]. In these tests, two stimuli are applied to the patient: (1) a test noxious stimulus and (2) a second conditioning painful stimulus applied in a distant (usually contralateral) region of the body. The test stimulus is done either at the same time or after the conditioning stimulus has ended [6]. In healthy individuals, the intensity of pain evoked by the test stimulus usually decreases with the application of the conditioning stimulus, echoing a healthy and fully functional descending inhibitory nociceptive system [7]. The effect of CPM in humans is long-lasting and may continue for up to 10 min [6]. The typology of stimuli used for DNIC/CPM testing is broad (e.g., thermal or mechanical). The nature of the test stimulus and conditioning stimulus may be of the same type (i.e., thermal conditioning and test stimulus), or there may be different modalities (i.e., thermal test stimulus and mechanical conditioning stimulus) [7,11]. Interestingly, the literature also indicates that it is possible to generate a DNIC/CPM response without a painful stimulus through the application of a strong, non-noxious stimulus that is easily detected by nociceptors [6,12,13]. In pre-clinical research, DNIC is evaluated in rodents in a similar form to that of clinical settings. The animals can be studied awake or in an anesthetized state depending on the aim of the experiment (e.g., for electrophysiology studies, animals must be anesthetized) [11]. In rats, the effect of DNIC may last for at least one hour [14]. Furthermore, DNIC/CPM responses are dependent on many interindividual factors [7,15], including age and gender [16,17], anxiety [18,19] and depressive [20] states, and genetic variations in the serotonergic *5-HTTLPR* long allele [21] and in the opioidergic allele *OPRM1 rs589046T* [22].

The results of CPM evaluation have been described as a prognostic tool for the development of chronic pain in healthy individuals and as a predictor of chronic pain outcomes in diseased patients [23]. The use of CPM as a prognostic biomarker for the development of chronic pain has been widely discussed and is a little controversial [24,25]. Nonetheless, Dursteler et al. (2021) showed that patients with low preoperative CPM analgesia have a higher probability of developing persistent pain after surgery [26]. In the same clinical context, Larsen et al. (2021) further demonstrated that impaired CPM analgesia may predict long-term postoperative pain even 12 months after surgery [27]. Despite these data, some clinicians question the prognostic validity of CPM as a predictor of nociceptive outcomes. Such controversy is related to the existence of different protocols for the CPM test with a wide variety of applied stimuli, which may cause a lack of consistency in the results [28]. Nonetheless, CPM was found to be reliably low in patients with chronic pain conditions, such as irritable bowel syndrome [29,30], migraine and tension-type headache [31,32,33], temporomandibular joint disorders [29,34], fibromyalgia [35], and osteoarthritis [36,37,38]. Additionally, Lewis et al. (2012) reported that approximately 70% of the studies comparing healthy individuals and patients with chronic pain demonstrated a significant reduction in CPM analgesia in patients [39]. Therefore, CPM may be a clinically significant parameter that may be added to the arsenal of diagnostic tools [28]. In addition to that, it may also be used as a therapeutic approach. Accordingly, a recent device harnessing CPM analgesia is currently being used for the treatment of migraines [40].

The potential of CPM as a useful predictor of the response to therapeutic treatment has been shown in several studies using duloxetine and tapentadol. The administration of the serotonin (or 5-hydroxytryptamine; 5-HT) and noradrenaline (NA) reuptake inhibitor duloxetine to patients with painful diabetic polyneuropathy revealed that a more efficient CPM predicted better drug efficacy. In the same study, CPM was improved and restored after treatment [41]. Similar observations were found with the analgesic tapentadol, which combines mu-opioid receptor agonism with NA reuptake inhibition [42]. As these drugs act on the descending modulatory system, CPM may also be viewed as an indicator of the integrity of this system. Thus, the assessment of CPM may guide clinical decisions and provide helpful information about the analgesic efficacy of a certain treatment according to the patient’s nociceptive profile [28].

Overall, the evidence supporting the relevance and usefulness of CPM in clinical settings appears to be solid. Although the number of pre-clinical studies focusing on the mechanisms that underlie DNIC has increased in recent years, this paradigm mostly remains understudied. In this review, we focused on the current state-of-the-art research regarding DNIC mechanisms in pre-clinical models of chronic pain. For an in-depth analysis, we will also reference human studies throughout the text. To correctly distinguish pre-clinical from clinical studies, we will use the term CPM when referencing studies in humans, DNIC when referencing pre-clinical studies, and DNIC/CPM when referencing both pre-clinical and clinical studies.

## 2. DNIC/CPM in Chronic Pain

Multiple studies have described the loss of efficiency of CPM/DNIC analgesia in long-term pain [6,7,11,28,39]. Neuropathic pain from various causes, such as chemotherapy-induced neuropathy [43] and spinal cord injury [44], has been associated with less efficient CPM. Patients with fibromyalgia present abnormalities in muscles or joints that are accompanied by severe pain and abnormal CPM [35,45]. Studies involving patients with osteoarthritis demonstrated that CPM is also lost in this condition [36,38,46]. Interestingly, following a pain-relief surgery and reassessment of these patients, the normal pressure pain modulation was reinstated, suggesting CPM had been restored [38]. A study simultaneously exploring CPM in irritable bowel syndrome and temporomandibular joint disorders showed increased sensitivity to heat pain and loss of CPM analgesia in these conditions [29]. In patients with diabetic polyneuropathy, migraines, and low back pain, the studies show a more complex scenario with mixed results [47,48,49,50]. Lower analgesic or even hyperalgesic CPM responses were found in patients with migraines compared to healthy controls [31,33,51]. Improved CPM analgesia was found with duloxetine treatment for migraines [52]. In diabetic polyneuropathy, while some studies found that CPM analgesia was restored by duloxetine [41] or tapentadol treatment [42], recent studies found discrepancies in CPM efficacy within different cohorts of patients [47,53]. In fact, less effective CPM was associated with a shorter chronic pain duration [53], and CPM analgesia was more efficient among patients with painful diabetic polyneuropathy when compared with those in which the disease does not elicit pain [47]. This is most likely caused by altered sensory input in the tested affected body regions, suggesting that the assessment of pain modulation in patients with neuropathy should be performed in intact sites [47]. In addition, other mechanisms might be at play. For example, duloxetine also acts as a potent inhibitor of transient receptor potential canonical 5 (TRPC5) calcium-permeable channels [54], which are expressed in human sensory neurons [55] and may be altered in the affected body regions in contrast to the intact sites. In patients with migraines, a variety of CPM paradigms have been applied with mixed results [49]. However, CPM protocols using cold stimuli as a heterotopically applied condition stimulus have revealed the most unanimous results [31,33,51]. This shows the importance of standardizing CPM protocols. Indeed, different methodologies have been used in the CPM/DNIC paradigms, including the location of the application of noxious stimuli and the type of test and conditioning stimuli, which often makes the comparison between protocols difficult [28]. In agreement, experts in the field have called for the standardization of CPM protocols in humans [56].

The few pre-clinical studies with rodent models of chronic pain show DNIC loss after pain chronification. Indeed, rats with early-stage monoiodoacetate-induced osteoarthritis presented normal DNIC, while at later stages of the condition, DNIC was abolished [57]. Our studies using the complete Freund’s adjuvant (CFA)-induced model of monoarthritis (MA) revealed a detailed temporal profile of DNIC analgesia progression. The DNIC analgesic responses were sustained from day 7 to day 21 of MA, peaked at day 28, and then progressively declined on days 35 and 42, eventually being completely lost at this later time point [58,59,60]. A previous study by Danziger et al. (1999) analyzed the progression of DNIC in CFA-induced MA, but the experimental period did not continue beyond 28 days of the disease [61]. Impairment of DNIC analgesia and/or loss of wide dynamic range (WDR) neuron inhibition has been reported in animals with peripheral [17,62,63,64,65,66,67] and central [68,69,70] neuropathic pain. This loss of DNIC seems to be associated with the chronification of pain and a subsequent imbalance between the descending facilitation and inhibition. This has been demonstrated by studies pharmacologically manipulating the monoaminergic and opioidergic systems, which indicates that these pain modulatory systems play a crucial role in DNIC.

## 3. DNIC and the Descending Modulation of Pain

### 3.1. Descending Pain Modulation

Descending pain modulation refers to the process by which the brain and spinal cord regulate the perception of pain. It involves a complex network of neural pathways that originate in the brain and extend down to the spinal cord, where they contribute to either enhancing or inhibiting the transmission of pain signals [71,72]. The most thoroughly understood descending modulatory pathways emanate from the periaqueductal gray area (PAG), the rostral ventromedial medulla (RVM), the *Locus coeruleus* (LC), and the dorsal reticular nucleus (DRt) [73,74]. Top-down modulation from the PAG is relayed by the RVM and the LC, with opioids playing a significant role in this process [73]. Descending pathways from the RVM can either inhibit or facilitate nociceptive transmission in the spinal cord. This bidirectional modulation is due to RVM neurons associated with pronociceptive ON- and antinociceptive OFF-cells. The LC also has a complex role in pain modulation, with both facilitatory and inhibitory effects on nociception; it facilitates pain through its ascending projections to various supraspinal areas [75,76,77,78] and inhibits pain through its descending projections to the spinal cord [79]. The DRt is a medullary area notable for its descending facilitation of nociceptive transmission through reciprocal excitatory connections with the spinal dorsal horn [80,81]. The DRt is also a relay for descending noradrenergic facilitation of pain from the LC [75,76,78,82]. The opioidergic system can directly and indirectly modulate the spinal-DRt-spinal circuitry [83,84,85]. This opioidergic modulation at the DRt is clinically relevant [74,86,87] and is involved in DNIC [4,5,14,59,60,65]. Several supraspinal areas, such as the prefrontal cortex and the amygdala, are linked to the emotional and cognitive components of pain and indirectly influence the regulation of the PAG-RVM circuit and LC through the opioidergic and monoaminergic systems. The modulation of the PAG-RVM circuit involves opioidergic influences from upstream brain structures, such as the prefrontal cortex, anterior cingulate cortex, and amygdala [74]. Specifically, this opioidergic influence is crucial for maintaining pain homeostasis [88,89]. Additionally, the functional dichotomy of the LC in pain modulation involves rostral projections to the anterior cingulate cortex and amygdala in the modulation of anxiety-like behaviors in rodents [79].

Opioids play a major role in top-down modulation from the PAG, the RVM, the LC, and the DRt. Remarkably, the PAG-RVM circuit is essential for the expression of μ-opioid receptor (MOR)-mediated analgesia through the disinhibition of γ-aminobutyric acid (GABA) interneurons [73,74,90]. In addition, at the RVM, opioids produce antinociception via direct inhibition of ON-cells, which express MORs, and indirect activation (i.e., disinhibition) of OFF-cells [91]. ON-cells have a well-established role in the maintenance of the sensory dimension of pain [92]. A recent study also found a crucial role for these cells in the maintenance of the affective dimension of chronic pain [93]. The neurochemical characterization of OFF- and ON-cells is starting to be uncovered. Neurons that functionally correspond to MOR-expressing ON-cells are GABAergic and project onto preproenkephalin-positive interneurons in the dorsal horn [94]. Neurons functionally corresponding to OFF-cells co-express GABA and preproenkephalin and directly project onto nociceptor terminals in the dorsal horn to inhibit nociceptive transmission [95]. Interestingly, a recent study using advanced methodologies suggested the existence of an excitatory monosynaptic pathway involving brain-derived neurotrophic factor (BDNF)-expressing neurons at the RVM connected to inhibitory spinal galanin-positive neurons [96]. The authors suggest that morphine-induced antinociception is mediated by this pathway rather than by the presynaptic inhibition of primary afferents [96].

In the LC, opioids have a bidirectional role in the control of the descending noradrenergic modulation. They produce antinociception through the disinhibition of NA neurons projecting to the spinal cord [74,97]. Opioids can also act through postsynaptic inhibitory (hyperpolarization) actions [98], and the hyperpolarization of LC neurons projecting to the spinal cord has been shown to produce hyperalgesia [99]. Additionally, opioids can suppress descending noradrenergic pain inhibition through a PAG-LC pathway [100]. Moreover, the effect of endogenous opioids in descending pain modulatory areas is also dependent on the opioid receptors (ORs) expressed therein [74]. Unlike delta (δ; DOR) activation, which yields effects similar to MORs, kappa (κ; KOR) and nociceptin (NOP) produce divergent effects [74]. A role for the different isoforms expressed from MORs has more recently been uncovered [74,87]. The opioidergic receptors are guanine nucleotide-binding (G)-protein-coupled receptors [101]. The canonical MOR isoform more often binds to inhibitory Gi proteins, which reduces neuronal activation and generates analgesia [74,101]. Contrastingly, MOR coupling to the stimulatory Gs protein shifts MOR signaling towards neuronal excitation, which has been associated with morphine-induced tolerance and hyperalgesia [74,102]. MOR coupling with the Gs protein is mediated through some MOR isoforms, such as C-terminal splice variants [102] and an N-terminally truncated 6-TM isoform [103]. The switch in MOR signaling to excitatory occurs in the PAG, where it is involved in tolerance [87,104]. At the DRt, the shift in MOR signaling from inhibitory to excitatory during chronic morphine infusion or chronic inflammatory pain contributes to opioid-induced hyperalgesia [87] and the switch from DNIC analgesia to hyperalgesia [60], respectively.

Bidirectional control of the spinal nociceptive transmission is also mediated through the release of NA and 5-HT from the LC and RVM, respectively. NA is released at the spinal cord and can have both inhibitory (antinociceptive) and facilitatory (pronociceptive) effects on pain transmission by acting through spinal alpha-2 adrenergic receptors (a2ARs) or alpha-1 adrenergic receptors (a1ARs) [105], respectively. A recent study also suggested inhibitory (antinociceptive) effects through the activation of a1ARs [106]. The release of 5-HT at the spinal cord may also have either an antinociceptive or a pronociceptive effect depending on the serotonergic receptors (5-HTRs) expressed [107,108]. The 5-HTRs are categorized into seven families: 5-HT1, 5-HT2, 5-HT3, 5-HT4, 5-HT5, 5-HT6, and 5-HT7. Among these, 5-HT1R, 5-HT2R, 5-HT3R, and 5-HT7R are involved in the nociceptive pathway. The 5-HT1R, coupled with the inhibitory Gi/o protein, reduces cyclic adenosine monophosphate (cAMP) levels, generating both anti- and pronociceptive effects. The 5-HT2R, coupled with the Gq/11 protein, increases inositol trisphosphate (IP3) and diacylglycerol (DAG) levels upon activation, resulting in an antinociceptive effect. The 5-HT7R, coupled with a stimulatory Gs protein, increases cAMP levels upon activation, producing both pro- and antinociceptive effects. Unlike the other receptors, the 5-HT3R is a ligand-gated cation channel that depolarizes the neuronal membrane when activated, causing antinociception but also maintaining painful stimuli [107]. Upon 5-HT release in a certain neuronal location, all serotonergic receptor types are activated simultaneously, instigating a mixture of excitatory and inhibitory regulatory effects.

The main objective of descending nociceptive modulation is to adjust the organism to the environment, and this is dependent on a constant balance between descending inhibition and facilitation [92]. In an acute stress response (fight or flight), for example, there is higher activation of NA neurons in the LC and an increase in both the synthesis and release of this neurotransmitter, which reduces the feeling of pain and fear and allows the individual to flee from distressing situations [109]. Moreover, in healthy individuals, the inhibitory pain mechanisms are usually more prominent than the facilitatory inputs, resulting in the attenuation of pain. DNIC analgesia likely reflects this homeostatic state. When this balance is disrupted towards pain facilitation, there is no alleviation of pain sensation, and chronic pain continues unabated [74,90,92]. During this event, the ongoing chronic noxious input may lead to many neuroplastic changes throughout the nervous system that inexorably foster the perfect environment for the onset of a chronic pain condition, negatively affecting the pain descending modulation mechanisms, which may be reflected by DNIC analgesia impairment.

### 3.2. DNIC Circuits Overlap with the Circuits Mediating Descending Pain Modulation

DNIC has been known for many years as an endogenous inhibitory paradigm. This phenomenon was first observed by Le Bars et al., who demonstrated that when DNIC was activated through the application of a heterotopic noxious conditioning stimulation, the electrophysiological activity of the spinal WDR neurons located in lamina V was depressed [4,5]. In these studies, Le Bars et al. reported that DNIC effects are exclusive for these types of convergent neurons since the application of a conditioning stimulus does not exert the same effect on noxious, non-noxious, and proprioceptive cells. Further studies also showed that DNIC mechanisms seem to occur post-synaptically at the spinal cord, as the effect of the application of spinal glutamate was strongly inhibited upon the presence of a conditioning stimulus [110]. In addition, DNIC can only be activated by noxious stimulation, as non-noxious stimuli did not inhibit the electrophysiological activity of the WDR neurons at spinal lamina V [4]. Interestingly, the involvement of A delta- or A delta- and C-peripheral fibers in DNIC was later confirmed in another study, where the pharmacological blockade of the spinal nerves conducting either the test stimulus or the conditioning stimulus decreased DNIC intensity [111]. As a result of these findings, the many studies that ensued focused on understanding the physiological implications and the possible neurochemical mechanisms behind the DNIC paradigm [26,35,57,62,63,70].

The potential circuitries that underlie DNIC analgesia have been a complete mystery since the first time this paradigm was reported. While initially it was believed that DNIC could be a phenomenon circumscribed to the spinal cord [4,5], it is now known that it involves “bottom-up” pathways that ascend through the ventrolateral funiculus [10,112,113] and return to the spinal cord through the dorsolateral funiculus [114]. The ascending projections of the superficial dorsal horn that are key in the transmission of DNIC information seem to rely on neurokinin-1 (NK1) receptors [115,116,117]. The NK1-positive neurons project to the parabrachial region [11,116,118], which in turn has projections to the PAG [119]. Spinal NK1-positive neurons are innervated by 5-HT3R fibers [120], suggesting that they are modulated by descending serotonergic inputs. The descending serotonergic modulation through 5-HT3Rs has been implicated in DNIC [59,63,70]. Our recent findings suggest a role for spinal 5-HT3Rs not only in the top-down modulation of DNIC but also in a bottom-up circuit, likely encompassing NK1-positive neurons, that is involved in the trigger of DNIC [59].

**Figure 1:**
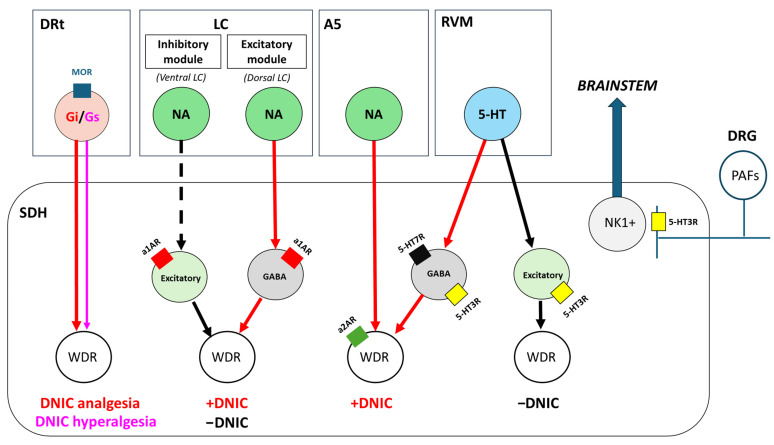
Proposed hypothetical circuits involved in the mediation of DNIC analgesia (red arrows; +DNIC), loss of DNIC analgesia (black arrows; −DNIC), and DNIC hyperalgesia (thinner purple arrows). In the *Locus coeruleus* (LC), two opposing circuits coexist. The first is an excitatory module, originating from dorsal LC neurons projecting to the spinal cord, which mediates DNIC analgesia. The second is an inhibitory module from ventral LC neurons projecting to spinal cord neurons (black dashed arrows), which abolishes DNIC. Both circuits exert their opposing effects through excitatory alpha-1 adrenergic receptors (a1ARs), likely located on excitatory or inhibitory (GABA) spinal cord interneurons, impinging on spinal wide dynamic range (WDR) neurons to mediate either the loss of DNIC or DNIC analgesia, respectively. In spite of the dichotomy of this circuit, functional studies emphasize the participation of the LC in DNIC analgesia. Therefore, the circuit mediating opposite effects in DNIC might contribute to adequately balancing the system in accordance with the organism’s needs. This again reflects the functioning of the descending pain modulatory system. In the A5 region, noradrenergic (NAergic) neurons projecting to the spinal cord contribute to DNIC analgesia by activating inhibitory alpha-2 adrenergic receptors (a2ARs), likely located on spinal WDR neurons. In the rostral ventromedial medulla (RVM), serotoninergic (5-HT) neurons project to either inhibitory GABAergic or excitatory spinal interneurons. Depending on the type of interneurons involved and receptors therein, this results in either DNIC analgesia or its abolishment. DNIC loss is probably mediated by the excitatory 5-HT3 receptor (5-HT3R) population that is most likely expressed in excitatory interneurons, while DNIC analgesia appears to be mediated by GABAergic interneurons that express both 5-HT3Rs and 5-HT7 receptors (5-HT7Rs). In this context, the effect of the 5-HT7R, which acts synergistically with the a2AR, is likely to become more prominent and mediate DNIC analgesia. Pre-synaptic excitatory 5-HT3Rs are also found in peripheral afferent fibers (PFAs) originating from dorsal root ganglia (DRG) neurons, which synapse onto projection neurons in the spinal cord expressing neurokinin-1 receptors (NK1+). These 5-HT3Rs are involved in a bottom-up circuit related to DNIC initiation. In the dorsal reticular nucleus (DRt), the coupling of mu-opioid receptors (MORs) to either the preferred/predominant inhibitory (Gi) proteins or the stimulatory (Gs) proteins, which are less recruited in physiological conditions (thinner purple arrow), determines whether DNIC analgesia or hyperalgesia occurs. This switch from inhibitory to excitatory signaling can disinhibit the descending facilitation from the DRt, contributing to the transition from DNIC analgesia, observed in physiological conditions, to hyperalgesia, as observed in chronic pain and prolonged opioid use. The concept of DNIC hyperalgesia challenges the established DNIC paradigm.

The involvement of supraspinal circuits in the mechanisms of DNIC was further supported by several other studies. Le Bars et al. showed that DNIC inhibition of WDR neurons was not observed in animals that underwent cervical transection [4,121,122]. Additionally, DNIC responses were not detected in tetraplegic animals or patients with sectioned spinal cords [113,122]. Further studies investigated the involvement of several brainstem, subcortical, and cortical areas. Villanueva et al. demonstrated that the thalamus is unlikely to be involved in DNIC circuitries, as both patients and animals with thalamic lesions exhibited no changes in DNIC [113,122,123]. The earlier studies investigating the involvement of some medullary and mesencephalic regions in DNIC have not always shown consensual data. While several works have reported that lesions of the PAG, parabrachial-cuneiform nucleus area, LC, and RVM are not directly responsible for significant changes in DNIC responses [124,125], pharmacological studies [126,127,128] showed different findings. Regarding the RVM, a more recent study demonstrated the involvement of OFF-cells of the nucleus raphe magnus in DNIC analgesia [129]. The A5 noradrenergic nucleus is involved in descending DNIC analgesia as well, as suggested by the work showing that DNIC responses were abolished upon inhibition of spinally projecting A5 neurons [130]. The involvement of the LC in DNIC analgesia is also indicated by recent studies where the lesion of the LC compromised DNIC analgesia in naïve animals [69]. Additionally, the chemogenetic stimulation of the LC restored the impaired DNIC analgesia in rats with traumatic brain injury [68], further emphasizing the participation of this area in the descending noradrenergic modulation of DNIC analgesia. Recent optogenetic studies suggest a more complex scenario [106] due to the modular organization of the nucleus [79]. In fact, Kucharczyk et al. (2022) showed that the optoactivation of the ventral LC’s module, which projects to the spinal cord [79], abolished DNIC, while a minor effect was obtained following optoactivation of the entire LC [106]. The authors suggest that the dorsal LC has either no effect or facilitates DNIC, while the ventral LC module inhibits DNIC [106]. Therefore, in the LC, two opposing circuits seem to coexist, one mediating descending inhibition of WDR neurons, reflecting DNIC analgesia, and a discrete LC-spinal circuit originated in a ventral cerulean neuronal population that abolishes DNIC (Figure 1).

The impact of the DRt on DNIC has also been recognized [80]. It was suggested by Le Bars et al. (2002) that DNIC triggered from the DRt could be part of a mechanism involved in the extraction of nociceptive information by depressing background body sensory activity, and therefore, descending inhibitory inputs from the DRt may constitute a separate type of inhibitory control [131]. Nonetheless, the DRt seems to be a crucial relay station of DNIC, acting through its direct and reciprocal projections to the spinal cord [112,132]. This circuitry is modulated by opioids acting locally on DRt spinally projecting neurons [14,60,65,84]. The DRt also seems to be a key ascending relay for the trigger of DNIC, namely through the activation of noradrenergic nuclei, as suggested by Kucharczyk et al. (2022) [106].

Several cortical and limbic regions, such as the anterior cingulate cortex (ACC) and amygdala, also influence DNIC responses [8,64,66,133,134]. For example, Navratilova et al. (2020) showed that injecting a MOR agonist into the right central nucleus of the amygdala restored DNIC in a neuropathic pain rat model [64]. These results, taken together with the fact that limbic areas are directly connected to DNIC-associated brainstem regions, such as the LC, RVM, and DRt, might account for the impact that psychological and emotional factors seem to have in the DNIC paradigm.

## 4. DNIC as a Descending Modulatory Mechanism: Neurochemical and Pharmacological Studies

Initial pharmacological studies implied the involvement of the descending opioidergic system in CPM/DNIC, while more recent work further reports the involvement of the noradrenergic and serotonergic components. The unraveling of the contribution of each of these neurochemical systems to DNIC has had some major advancements over the past decades. The involvement of monoamines and opioids, together with that of other neurotransmitters, has been reviewed in pre-clinical [11] and clinical studies [135]. In the next section, we thoroughly analyze the latest data on the monoaminergic and opioidergic studies, focusing particularly on pre-clinical models of chronic pain, and integrate these data in light of the functioning of the descending modulatory circuit (Figure 1).

### 4.1. Descending Serotonergic System

Early studies showed the importance of the serotonergic system in DNIC by revealing that either the depletion of 5-HT [128] or blockade of 5-HT receptors [126] strongly decreased the inhibitory effects of DNIC upon WDR neuron activity, while a precursor of 5-HT potentiated it [126]. However, the specific contribution of this descending system to DNIC has been quite hard to unravel due to the wide variety of existing 5-HTRs, which can produce simultaneous facilitatory and inhibitory effects upon neuronal activity [90,107]. Additionally, the neurochemical studies evaluating the contribution of the serotonergic system to DNIC have focused mainly on the 5-HT3R and 5-HT7R types. The main studies are presented in Table 1. The studies in healthy humans revealed a less conclusive involvement of the monoaminergic system in CPM [135]. However, in pathological chronic pain conditions where the descending pain modulatory system is impaired, which is reflected by a defective CPM analgesia [7], the available studies show a less ambiguous scenario. Indeed, in patients with diabetic polyneuropathy and migraines, treatment with a 5-HT and NA reuptake inhibitor (duloxetine) improved CPM analgesia [41,52]. Similar findings were obtained when duloxetine was administered systemically in pre-clinical models of osteoarthritis and peripheral or central neuropathic pain [67,69,70]. Concerning the effects of selective 5-HT reuptake inhibitors (SSRIs), spinally administered citalopram and fluoxetine restored DNIC in a peripheral neuropathic pain model, while their systemic application yielded no results [62]. Thus, the data suggest that the effect of SSRIs on DNIC may potentially be dependent on the administration route and highlight the importance of specifically targeting spinal 5-HT receptors to restore DNIC. The roles of spinal 5-HT7R and 5-HT3R in DNIC analgesia have been the most well-studied until the present day [57,59,62,63,70,136]. In electrophysiological studies performed in the monoiodoacetate-induced osteoarthritis rat model, the blockade of spinal inhibitory 5-HT7Rs reduced DNIC in the early stages of this condition, while the activation of these receptors restored DNIC during the chronic stages [57]. The restoration of DNIC by citalopram and fluoxetine in the peripheral neuropathic pain model by Bannister et al. (2017) was reversed by 5-HT7R antagonism [62]. This work suggests that 5-HT might exert an inhibitory action upon WDR neurons through the activation of the 5-HT7R, thus contributing to DNIC analgesia. Further confirming this hypothesis, DNIC analgesia was reversed by a 5-HT7R antagonist in naïve rats [136]. However, the serotonergic input seems to act synergically with and be dependent on the noradrenergic input to restore DNIC analgesia since the blockade of spinal a2ARs prevents DNIC restoration by SSRIs, at least following peripheral neuropathic pain [62]. The neurochemical data regarding the 5-HT7R are scarce. The Lockwood et al. (2019) study in osteoarthritic rats showed that the levels of these receptors were unchanged in both the dorsal horn and dorsal root ganglia when DNIC was lost [57]. Regarding the 5-HT3R, our studies showed that the blockade of the excitatory spinal 5-HT3R restored DNIC analgesia in a model of CFA-induced monoarthritis [59]. In agreement, following 5-HT3R antagonism, the inhibition of WDR neurons was increased in naïve animals and restored in a rat model of peripheral neuropathy [63]. In the chronic joint inflammatory pain model, we also observed an increased expression of 5-HT3Rs at a time of loss of DNIC analgesia. In this same study, an upregulation of spinal 5-HT was also found, together with increased serotonergic activity evaluated by labeling phosphorylated extracellular signal-regulated protein kinases 1 and 2 (pERK1/2) at the RVM [59]. This increased serotonergic activation at the RVM, paralleled by an upregulation of spinal 5-HT, has been previously reported in a rat model of neuropathic pain [137]. In contrast, in a model of traumatic brain injury, neither the antagonism of spinal 5-HT3Rs [70] nor the spinal depletion of serotonergic fibers [69] restored DNIC. Additionally, in this model, increased levels of spinal 5-HT were found [70], and the systemic administration of escitalopram restored DNIC [69,70]. Few studies have been conducted to explore the role of the other 5-HTRs in DNIC. In naïve rats, the inhibitory effects of DNIC upon WDR neuron activity were diminished by the blockade with metergoline, which acts upon several 5-HTR subtypes, including 5-HT1R, 5-HT2R, 5-HT6R, and 5-HT7R [126]. In a pioneering work establishing a behavioral correlate for DNIC, the authors found that antagonizing 5-HT2Rs with cinanserin blocked the potentiation of DNIC analgesia produced by a 5-HT precursor [138]. These results agree with a strong reduction in the inhibitory effects of DNIC on WDR neuron activity produced by cinanserin [126]. The antagonism of 5-HT1ARs in naïve animals reduced DNIC analgesia [139]. In a model of medication overuse-induced migraines, continuous exposure to a high dose of the 5-HT1R agonist sumatriptan followed by noxious stimulation induced the loss of DNIC analgesia, while a low dose had no effect [140].

The serotonergic input is involved in DNIC analgesia through 5-HT7Rs [62,136]. The role that 5-HT7Rs might play in DNIC is consistent with their location in the spinal cord, where they are mainly expressed postsynaptically in local interneurons of the superficial dorsal laminae and presynaptically in peptidergic fibers [107]. Additionally, immunocytochemical studies showed 5-HT7R co-localization with GABAergic neurons at the spinal dorsal horn [141]. Thus, given the excitatory action of 5-HT7Rs [107], they might exert an inhibitory action upon WDR neurons through the activation of spinal inhibitory GABAergic interneurons. Additionally, in chronic inflammatory and peripheral neuropathic pain models, the serotonergic input contributes to abolishing DNIC analgesia via the activation of 5-HT3Rs. At the spinal cord, 5-HT3Rs are in presynaptic terminals and postsynaptic interneurons of the superficial dorsal horn layers [107]. Postsynaptically, 5-HT3Rs are positioned in inhibitory GABAergic interneurons, through which they exert antinociceptive effects [142], and in excitatory interneurons and terminal fibers apposing onto spinal NK-1 projection neurons [120]. The location of 5-HT3Rs in excitatory interneurons and terminal fibers is likely responsible for facilitating nociceptive responses of some dorsal horn neurons [143,144]. Furthermore, the ascending nociceptive circuit is composed of NK1-expressing neurons [116,118], which are also involved in triggering DNIC [116]. These NK1+ neurons are innervated by either dense or sparse 5-HT3R fibers [120]. In MA, the increased basal levels of 5-HT observed at 42 days, along with heightened 5-HT3R expression [59], might cause a shift in the recruitment of the differentially 5-HT3R-innervated NK1 neurons, thus contributing to the maintenance of persistent pain [107]. Considering the pronounced 5-HT3R pronociceptive effects, how can one reconcile the restitution of DNIC by SSRIs? This can only be explained if the inhibitory effect of the 5-HT7Rs becomes more prominent, thus restoring DNIC [57]. Though the 5-HT3R might indeed play a crucial facilitatory role in the ablation of DNIC analgesia in chronic pain, other mechanisms are also at play. Studies in the brain indicate that SSRI action upon 5-HT3Rs often involves modulation and inhibition of these receptors rather than their desensitization [145,146]. In traumatic brain injury, the serotonergic input to the spinal cord does not seem to contribute to abolishing DNIC analgesia via spinal 5-HT3Rs, as their blockade fails to restore DNIC [70]. The reduced sensitivity of a2ARs is more likely to be responsible for the loss of DNIC [70]. The increased spinal levels of 5-HT in this central neuropathic model [70], in contrast, contribute to restoring DNIC. However, the imbalance caused by the impairment of a2AR sensitivity does not seem to be offset by an increase in spinal 5-HT. Whether or not the 5-HT7R is implicated in this lack of 5-HT effect is not known, as the studies on traumatic brain injury did not target this receptor type.

In summary, the serotonergic system is involved in DNIC through 5-HT neurons projecting from the RVM to either inhibitory GABAergic or excitatory spinal interneurons. Depending on the type of interneurons involved and receptors therein, this results in either DNIC analgesia or its abolishment. DNIC loss is probably mediated by the excitatory 5-HT3R population that is most likely expressed in excitatory interneurons, while DNIC analgesia appears to be mediated by GABAergic interneurons that express both the 5-HT3R and 5-HT7R. In this context, the effect of the 5-HT7R, which acts synergistically with the a2AR, is likely to become more prominent and mediate DNIC analgesia. Additionally, pre-synaptic excitatory 5-HT3Rs expressed in afferent fibers may also be involved in a bottom-up circuit related to DNIC initiation (Figure 1).

### 4.2. Descending Noradrenergic System

The contribution of the descending noradrenergic system to DNIC analgesia has been very well studied both neurochemically and pharmacologically. The different studies showcase the functional relevance of the effects of descending noradrenergic inhibition, mostly through spinal a2ARs. Moreover, they indicate that a potential impairment of this modulatory system has an impact on DNIC analgesia extinction in chronic pain. Table 1 summarizes the main recent studies that focused on the noradrenergic system. DNIC is significantly attenuated by the a2AR antagonists in normal healthy animals [57,63,67,69,70,130,136,139,147]. In electrophysiological studies, where DNIC is evaluated as the inhibition of WDR neurons, the blockade of spinal a2ARs also abolished DNIC in rats with early-stage osteoarthritis [57]. The activation of spinal a2ARs restored DNIC that had been lost in osteoarthritic rats at a late stage of the disease [57]. Additionally, intrathecal reboxetine, a selective NA reuptake inhibitor, and tapentadol, which is a dual MOR agonist and NA reuptake inhibitor, reinstated DNIC in rats with peripheral neuropathic pain [63] and late-stage osteoarthritis [57]. Concomitantly, with the loss of DNIC analgesia at the late stages of chronic joint monoarthritis, we found no changes in the spinal a2AR protein levels [58]. Coincidentally, the mRNA expression of the receptor remained unchanged at the spinal dorsal horn and lumbar dorsal root ganglia in the late stage of osteoarthritis induced by monoiodoacetate [57]. Moreover, we found a downregulation of spinal NA along with increased spinal levels of dopamine beta-hydroxylase (DBH) and increased neuronal activity in the LC at the same time point of the disease [58]. This suggests that activation of the descending noradrenergic system likely compensates for the increased need for spinal NA by recruiting the biosynthetic machinery [58]. These findings also indicate that there is a counteracting attempt to regain DNIC analgesia at the spinal level during prolonged stages of chronic pain. However, the compensatory mechanisms may not always occur through significant changes in the expression of the receptor but rather in its functionality. In accordance with the CFA model of chronic joint inflammatory pain, we found that the spinal a2ARs were potentiated when DNIC analgesia was abolished [58]. In addition to this spinal compensatory mechanism, other supraspinal events may be involved. Indeed, we also observed increased levels of neuronal activity evaluated by labeling pERK1/2 in areas associated with the processing of the emotional component of pain, such as the basolateral amygdala and the ACC [58], that project to and receive projections from the LC [148,149].DNIC analgesia was also found impaired in central neuropathic pain induced by traumatic brain injury [68,69,70]. In this model, no significant differences in the levels of spinal NA were observed, but the spinal a2R sensitivity was reduced [70]. This may explain why systemic administration of reboxetine failed to restore DNIC analgesia in these animals [69,70].

The contribution of alpha adrenoreceptors to the inhibition of WDR neurons seems to differ according to the noradrenergic cell group involved. In fact, recent work by Kucharczyk et al. shows that the optoactivation of either the A5 or the LC results in the inhibition of WDR spinal neurons through a2ARs [130] or a1ARs [106], respectively. Given the opposite effects of both receptors on neuronal excitability, the effect of a1ARs is likely indirectly mediated through GABAergic inhibitory interneurons [150,151,152]. During the application of the DNIC paradigm, it is well established that the inhibition of WDR neurons is mediated through a2ARs [11,130]. The direct A5-spinal cord projection seems to play a key role in the mediation of this effect [130]. In the LC, the recently shown inhibitory and facilitatory modules for DNIC regulation seem to mediate their effects through a1ARs [68,106]. Given the excitatory action of the a1AR, this apparently surprising effect can only be explained by the localization of the receptor in dichotomic neuronal populations. In addition to its putative action on GABAergic inhibitory interneurons, electrophysiological and pharmacological data also suggest that the a1AR can enhance the activity of both excitatory interneurons and projection neurons of the spinal dorsal horn [72,153].

In summary, the noradrenergic contributions to DNIC seem to occur from the LC and the A5 cell group. In the LC, two opposing circuits coexist. The first is an excitatory module originating from dorsal LC neurons projecting to the spinal cord, which mediates DNIC analgesia. The second is an inhibitory module from ventral LC neurons projecting to spinal cord neurons, which abolishes DNIC. Both circuits exert their opposing effects through excitatory a1ARs, likely located on excitatory or inhibitory (GABA) spinal cord interneurons, impinging on WDR neurons to mediate either the loss of DNIC or DNIC analgesia, respectively. In the A5 region, noradrenergic neurons projecting to the spinal cord contribute to DNIC analgesia by activating inhibitory a2ARs, likely located on spinal WDR neurons (Figure 1).

### 4.3. Descending Opioidergic System

The opioidergic contribution to DNIC mechanisms was one of the very first findings that emerged in the initial studies with the paradigm in clinical trials [154,155]. However, the subsequent studies in humans are divisive on establishing the exact role of the descending opioidergic system in DNIC, as reviewed recently [135]. This emphasizes the complexity of the mechanisms entailing the participation of the opioidergic system in DNIC. In rodents, this association has been reported multiple times (Table 1). In initial electrophysiological studies, it was reported that systemic and intracerebroventricular morphine inhibits DNIC [1,2,156] and that this effect was reversed by the administration of the opioid receptor antagonist naloxone [1,156]. The systemic injection of naloxone partially reduced DNIC [3]. The effects of naloxone found in electrophysiological studies were also behaviorally confirmed [138]. This pioneering work provided a clue about the involvement of the opioidergic system in DNIC. Later studies showed that systemic naloxone was able to revert DNIC analgesia, but this effect was dependent on the conditioning stimulus [147]. In painful conditions such as acute inflammation, systemic naloxone was also shown to prevent DNIC analgesia [14]. In contrast, the systemic administration of a specific KOR antagonist prevented the loss of behavioral DNIC analgesia in female rats with chronic orofacial pain [17]. In a model of medication overuse-induced migraines, continuous exposure to morphine abolished DNIC analgesia [140]. Altogether, these studies highlight the complexity of the opioidergic involvement in DNIC.

More recent studies have focused on a more targeted approach to the pharmacological administration of opioidergic receptor agonists and antagonists, with the injection of these drugs in specific opioidergic-modulated supraspinal nuclei, such as the DRt, RVM, and amygdala. Intra-DRt injection of naloxone blocked DNIC in sham rats [65]. Additionally, we found that DAMGO, a mu-opioid receptor agonist, at the DRt increased DNIC analgesia in normal, healthy animals [60]. These studies reflect not only that DRt descending pathways are involved in DNIC but also that these mechanisms require opioidergic signaling through MORs. Intra-DRt naloxone blocked DNIC analgesia in acute muscle pain [14] but did not produce any effects on DNIC in animals that had lost DNIC following spinal nerve ligation-induced neuropathic pain [65]. In chronic joint pain, we have shown that the activation of MORs at the DRt produced a hyperalgesic effect (i.e., shifted DNIC analgesia to hyperalgesia) [60]. These latter findings suggest differential opioidergic signaling at the DRt in acute [14] vs. chronic phases of inflammatory pain [60]. Additionally, the role of MOR-mediated modulation of DNIC at the DRt might differ in situations where DNIC is lost during inflammatory [60] or neuropathic pain [65]. Regarding the RVM, an early study showed that MOR activation at the Raphe Magnus has no effect on DNIC when evaluated by the activity of spinal trigeminal nucleus oralis convergent neurons [157]. In agreement, a later study showed that naloxone in the RVM had no effect on DNIC analgesia in a model of acute muscle pain [14]. These results are puzzling, given the importance of the opioidergic modulation of ON- and OFF-cells in the RVM [74,91]. In addition, the inactivation of the RVM restored DNIC analgesia that had been lost following continuous exposure to morphine in a model of medication overuse-induced migraines [158]. Therefore, there may be opioid involvement in the modulation of DNIC by the RVM.

The opioidergic system also modulates DNIC through its action on MORs and KORs at the amygdala. In fact, either MOR activation [64] or KOR blockade [66] at the central nucleus of the amygdala restored DNIC analgesia [64] as well as the inhibition of WDR neurons [66] in a neuropathic pain rat model. Interestingly, both MOR and KOR signaling are involved in the modulation of the aversive/affective dimension of neuropathic pain and DNIC [64,159]. Therefore, DNIC is modulated by supraspinal areas involved in the affective component of pain, and this is mediated by opioid signaling. Interestingly, we found a loss of DNIC that was concomitant with anxiodepressive-like behaviors and neuronal activation of supraspinal areas involved in the affective component of pain, including the amygdala, in a chronic pain model [58]. The effect that opioids have on DNIC, specifically at the spinal cord level, has been poorly investigated. However, the studies with tapentadol, through its effects on the opioidergic component, are perhaps the most suggestive of the opioidergic spinally mediated mechanisms. Tapentadol, which acts simultaneously as a MOR agonist and an NA reuptake inhibitor [42], restored DNIC in late-stage osteoarthritis [57] and spinal nerve ligation [63]. The effects of tapentadol are mostly attributed to a synergistic effect of MOR activation and inhibition of NA reuptake in the spinal cord [160]. Consistent with this effect, MORs are expressed in the spinal cord, where they serve as an interface for ascending inhibition and descending opioidergic inhibition triggered from the PAG-RVM circuitry [74,161]. Indeed, endogenous opioid peptides are released from descending fibers, arising from the PAG-RVM circuitry, into the spinal cord [94,95]. The role of spinal MORs in the mediation of descending opioidergic inhibition is further corroborated by the conditional knockout of mu-opioid receptors in primary afferent neurons, which significantly reduced the analgesic effect induced by the activation of the PAG-RVM circuit [162].

The available molecular studies regarding the involvement of the opioidergic system on DNIC have been focused on the DRt. Recent studies have shown that during opioid-induced hyperalgesia, MOR activity at the DRt switches its coupling with the inhibitory Gi proteins to excitatory Gs proteins, causing an upregulation in the cAMP response element-binding protein (CREB) phosphorylation, which accounts for the hyperalgesia effects observed upon MOR activation [87]. In the CFA model of chronic joint pain, we also found increased levels of phosphorylated CREB (pCREB) at a time point of disease evolution when DNIC analgesia is lost and when the activation of MORs at the DRt produces DNIC hyperalgesia [60]. This effect is blocked by pretreatment with an ultra-low dose of naloxone [60], which inhibits MOR coupling with the stimulatory Gs protein and restores its coupling with the inhibitory Gi [163]. This further reinforces the occurrence of a probable shift of MOR signaling at the DRt, which may likely contribute to the extinction of DNIC analgesia [60].

These studies, together with previous work evaluating MOR signaling at the DRt [86], indicate that in physiological conditions, the opioidergic input to the DRt is necessary for the expression of DNIC analgesia. Thus, DNIC analgesia relies on the inhibition of descending facilitation from the DRt. This is also consistent with the inhibitory effects of MORs on neuronal excitability. Indeed, MOR activation at the DRt induces the intracellular coupling of these receptors with inhibitory Gi proteins, inhibiting the adenylyl cyclase and producing an analgesic effect [86]. In a neuropathic pain condition in which DNIC is lost, and the MOR blockade does not alter the DNIC outcome [65], the tonic inhibitory opioidergic input is lost. This is likely due to the desensitization of MORs that occurs in neuropathic pain [86]. In chronic inflammatory pain, the switch in MOR signaling to excitatory disinhibits the descending facilitation from the DRt, thus contributing to switching DNIC from analgesia to hyperalgesia. Our results also likely uncovered a facilitatory pathway for DNIC, which has previously been postulated [136]. This pathway originates in the DRt and terminates in lamina V of the spinal dorsal horn [80,81], where WDR neurons are located and whose activity is enhanced by DRt activation [164].

In conclusion, although the opioidergic system was the very first to be identified as being implicated in DNIC, its role remains mostly controversial due to the intricacies of the pathways involved. One of the brain regions where the opioidergic contribution to DNIC has been more explored is the DRt. In this nucleus, the coupling of MORs with either inhibitory or stimulatory G proteins determines whether DNIC analgesia or hyperalgesia occurs. This switch from inhibitory to excitatory signaling can disinhibit the descending facilitation from the DRt, contributing to the transition from DNIC analgesia, observed in physiological conditions, to hyperalgesia, as observed in chronic pain and prolonged opioid use (Figure 1).

**Table 1:** Summary of recent pharmacological studies performed in animal models of pain.

	5-HT STUDIES	NA STUDIES	OPIOID STUDIES
**Normal healthy** **animals**	5-HT3R blockade increases DNIC analgesia magnitude and WDR inhibition [59,63] 5-HT7R blockade abolishes DNIC analgesia [136] Spinal 5-HT1AR antagonism reduced DNIC analgesia [139]	a2AR blockade attenuates/abolishes DNIC analgesia and WDR inhibition [57,63,67,69,70,130,136,139,147] LC lesion (neurotoxin) abolishes DNIC analgesia [69] LC chemogenetic activation produces DNIC analgesia [68] A5-SC optoinhibition abolishes DNIC (WDR neuronal inhibition) [130] LC:SC optoactivation abolishes DNIC (WDR neuronal inhibition) through a1ARs [106]	Systemic naloxone reverses DNIC analgesia induced by chemical but not electrical conditioning stimuli [147] Systemic and intracerebroventricular naloxone reduced DNIC analgesia [139] MOR activation at the DRt increases DNIC analgesia [60] Naloxone at the DRt abolishes DNIC [65]
**INFLAMMATORY PAIN**			
**Muscle inflammation** DNIC was enhanced and similar in acute and chronic phases of inflammation			Systemic naloxone abolished DNIC analgesia Naloxone in the DRt abolished DNIC analgesia Naloxone in the RVM had no effect on DNIC analgesia [14]
**Early-stage osteoarthritis** **(Monoiodoacetate model)**	Blockade of spinal 5-HT7Rs partially reduced DNIC (WDR neuronal inhibition) [57]	Blockade of spinal a2ARs abolished DNIC (WDR neuronal inhibition) [57]	
**Late-stage osteoarthritis** **(Monoiodoacetate model)** Loss/attenuation of DNIC analgesia or WDR neuronal inhibition	Activation of spinal 5-HT7Rs restored DNIC (WDR neuronal inhibition) [57]	Activation of spinal a2ARs restored DNIC (WDR neuronal inhibition) [57]	
	5-HT7R levels were unchanged in the dorsal horn and lumbar dorsal root ganglia [57]	a2AR levels were unchanged in the dorsal horn and lumbar dorsal root ganglia [57]	
	Duloxetine improved DNIC analgesia [67]		
		Tapentadol restored DNIC (WDR neuronal inhibition) [57]	
**Intermediate stage of monoarthritis** **(CFA model)** Magnitude of DNIC analgesia peaked at an intermediate time point		No changes in spinal levels of DBH No changes in spinal NA levels [58]	
**Late-stage monoarthritis** **(CFA model)** Loss of DNIC analgesia	Increased spinal 5-HT levels [59] Blockade of spinal 5-HT3Rs restored DNIC analgesia [59] Increased spinal 5-HT3R expression [59] Increased RVM serotonergic activity (pERKs1/2 + TPH labeling) [59]	Decreased spinal NA levels Increased spinal levels of DBH [58] Spinal a2ARs potentiated [58] No changes in the spinal a2AR expression [58] Increased LC neuronal activity (pERKs1/2 labeling) Increased neuronal activity (pERKs1/2 labeling) in brain areas connected with the LC involved in the affective component of pain [58]	MOR activation at the DRt produces DNIC hyperalgesia Blockade of MORs coupling with the excitatory Gs protein at the DRt restores DNIC analgesia [60] Decreased levels of MORs and increased pMORs at the DRt Increased levels of pCREB at the DRt [60]
**PERIPHERAL NEUROPATHY**			
**Spinal nerve ligation** Loss of DNIC analgesia and WDR neuronal inhibition	5-HT3R blockade restored DNIC (inhibition of WDR neurons) [63] Systemic citalopram and fluoxetine yielded no results Spinal application of citalopram and fluoxetine restored DNIC (inhibition of WDR neurons) through the 5-HT7R and a2AR [62].	Reboxetine restored DNIC (inhibition of WDR neurons) [63]	Naloxone in the DRt had no effects on DNIC inhibition of WDR neurons [65] Systemic KOR blockade restored DNIC analgesia [66] KOR blockade at the central nucleus of the amygdala restored DNIC analgesia and WDR neuronal inhibition [66] Morphine at the ipsilateral central nucleus of the amygdala restored DNIC analgesia [64]
			Systemic KOR blockade prevented the loss of DNIC analgesia [17]
**Partial sciatic nerve ligation** Attenuation of DNIC analgesia	Duloxetine improved DNIC analgesia [67]		
**Chronic constriction injury of the infraorbital nerve** Loss of DNIC analgesia in females		Tapentadol restored DNIC (inhibition of WDR neurons) [63]	
**TRAUMATIC BRAIN INJURY** DNIC analgesia impaired	Spinal depletion of 5-HT fails to restore DNIC [69]	LC chemogenetic activation restores DNIC through a1ARs [68]	
	5-HT3R blockade fails to restore DNIC [70]	Reduced spinal a2AR sensitivity [70]	
	Systemic escitalopram restores DNIC [69,70] Escitalopram restores DNIC; α2AR signaling is not involved [69] Increased spinal 5-HT levels [70]	Reboxetine fails to restore DNIC analgesia [69,70] Unchanged spinal NA levels [70]	
	Duloxetine restores DNIC [69,70]		
**MEDICATION OVERUSE-INDUCED** **MIGRAINES** Loss of DNIC analgesia/inhibition of medullary dorsal horn neurons	Continuous exposure to a low dose of the 5-HT1R agonist sumatriptan did not cause loss of DNIC analgesia [140] Continuous exposure to a high dose of sumatriptan followed by noxious stimulation induced loss of DNIC analgesia two weeks after treatment cessation [140]		Continuous exposure to morphine caused opioid-induced hyperalgesia (OIH) and abolished DNIC analgesia both during and upon cessation of OIH manifestation [140] Continuous exposure to morphine abolished DNIC, and inactivation of the RVM restored DNIC [158]

Legend: 5-HT: 5-hydroxytryptamine (serotonin); 5-HT3R: 5-hydroxytryptamine receptor type 3; 5-HT7R: 5-hydroxytryptamine receptor type 7; a1AR: alpha adrenergic receptor type 1; a2AR: alpha adrenergic receptor type 2; CFA: complete Freund’s adjuvant; CPM: conditioned pain modulation; DBH: dopamine beta-hydroxylase; DNIC: diffuse noxious inhibitory control; DRt: dorsal reticular nucleus; KORs: κ-opioidergic receptors; LC: *Locus coeruleus*; MORs: μ-opioidergic receptors; NA: noradrenaline; pCREB: phosphorylated cyclic-AMP response element-binding protein; pERKs1/2: phosphorylated extracellular signal-regulated protein kinases 1 and 2; pMORs: phosphorylated μ-opioidergic receptors; RVM: rostral ventromedial medulla; TPH: tryptophan hydroxylase; WDR: wide dynamic range.

## 5. Is DNIC Only a Descending INHIBITORY/ANALGESIC Mechanism?

DNIC is a paradigm very commonly known for its endogenous analgesic nature. However, there is mounting evidence that DNIC responses may not always be analgesic. Indeed, the paradigm of CPM in humans can manifest as both hyperalgesia and analgesia [28,165,166,167]. Recent pre-clinical studies also suggest this duality. Two pathways emanating from the LC that play opposing roles in DNIC seem to coexist [106]. The variability of spinal 5-HTRs and their effects on neuronal excitability may also contribute to the dual outcomes of the serotonergic pathways observed in DNIC. Additionally, Tansley et al. reported that the outcome of DNIC stimulation is dependent on the intensity of the test stimulus given to awake animals, such that the paradigm generated hyperalgesia with lower-intensity stimuli and analgesia with stronger stimulation [168]. In view of the later observations showing opposite effects in DNIC behavioral responses, some authors have suggested a review of DNIC nomenclature. Bannister et al. proposed that DNIC should refer only to the mechanistic changes observed in anesthetized animals, specifically indicating the inhibition of WDR neurons after a conditioning stimulus. Moreover, the term “descending control of nociception” (DCN) was suggested as being a better nomenclature for the behavioral correlate of DNIC in awake animals in order to reflect the analgesic and hyperalgesic effects [165].

The duality of the DNIC/DCN nature brings a new set of unanswered concerns. Indeed, most pre-clinical studies show an ablation of DCN/DNIC analgesia in animals with chronic inflammatory or neuropathic pain. This is translated into a decrease in the intensity of response or the total absence of DCN/DNIC analgesia when compared to control groups. Considering the possibility of the existence of a hyperalgesic DCN, it is feasible to question if this absence of DCN/DNIC analgesia is, indeed, the real output of the behavioral evaluation of DNIC. Instead, it could reflect a methodological limitation of the tools used so far to measure the variation in DCN/DNIC magnitude. If the latter hypothesis is correct, then a serious review of all nociceptive behavioral assessment tools must be performed in order to determine which methods are the most adequate to detect lower or negative variations during the nociceptive behavioral evaluation of DCN/DNIC.

Nemoto et al. suggested that opposing neurochemical pathways may mediate the hyperalgesic and analgesic DCN [136]. Our recent findings suggest that the DRt, through its opioidergic regulation, may be involved in mediating both the hyperalgesic and analgesic DNIC/DCN and that these processes might be dependent on different molecular intracellular mechanisms. The branch of the DRt that projects to the spinal laminae V [80,81] and controls the activity of the WDR neurons [164] likely mediates the DRt effects on DCN/DNIC. Supporting this, the tonic opioidergic inhibition of this branch, in normal conditions, allows the electrophysiological expression of DNIC [65]. Behaviorally, we showed that DCN/DNIC analgesia in healthy animals is enhanced when a selective agonist activates MORs located in the DRt [60]. At late-stage monoarthritis, we also found that MOR activation at the DRt causes DCN/DNIC analgesia to become hyperalgesic. This was due to a switch in MOR signaling at the DRt from inhibitory to excitatory, likely increasing the descending facilitation from the DRt [60]. This change may also be responsible for the extinction of DCN/DNIC analgesia in late-stage monoarthritis [60], which also happens in chronic pain patients [6,7,11,28,39].

## 6. Conclusions

DNIC is a mirror of descending modulation, encompassing both inhibitory and excitatory effects. Under normal conditions or acute pain, the inhibitory pathway is predominant, reflecting the analgesic nature of DNIC, which in turn indicates a balanced functioning of descending modulation. In this context, facilitatory pathways, such as those emanating from the DRt, are likely silenced by an opioidergic input. In chronic pain, the imbalance in descending modulation towards increased facilitation disinhibits/enhances these facilitatory pathways. One of the mechanisms through which these facilitatory pathways become disinhibited entails the shift in opioidergic signaling at the DRt. Depending on the magnitude or nature of this disinhibition of the facilitatory effects, the outcome of DNIC may result in either a loss of analgesia or the development of hyperalgesia.

The translational value of this paradigm suggests that assessing CPM in patients with pain could enable us to predict the effectiveness of certain drugs, offering a potentially valuable tool for determining the likelihood of treatment success. In fact, although opioids still provide the strongest analgesia not met by any other drugs/compounds, their use may not always be ideal because both chronic pain and chronic treatment with opioids may alter MOR signaling in facilitatory pathways. This results in the transformation of DNIC effects from analgesia, or lack of analgesia, to hyperalgesia, reflecting the maladaptation of the descending modulatory system. Therefore, opioid-based therapy may be counterproductive since it may likely exacerbate this effect in some chronic pain conditions. At the DRt, MORs are also prone to alterations such as downregulation, desensitization, and increased degradation during chronic neuropathic pain [74,86]. In addition, MORs may suffer additional alterations, such as signaling bias towards beta-arrestins responsible for adverse side effects of opioid drugs. Adding another layer of complexity, MOR agonists may also display a differential preference for the recently described isoforms of the receptor. These isoforms also have isoform-specific biased signaling, not only towards beta-arrestins vs. G-proteins but also regarding the recruitment of stimulatory (Gs) vs. inhibitory (Gi) proteins [74]. All these alterations are likely to affect the functioning of the descending pain modulatory system. Therapeutic approaches targeting only a2ARs may also be challenging due to the unwanted cardiovascular side effects of a2R agonists, which might compromise their usefulness in chronic pain relief. On the other hand, serotonin and norepinephrine reuptake inhibitors (SNRIs) may represent a better therapeutic option. First, the effects of SSRIs such as citalopram and fluoxetine on DNIC have been shown to be mediated through 5-HT7Rs [62,136]. Second, the blockade of spinal a2ARs prevents DNIC restoration by the same SSRIs [62]. These studies indicate that inhibiting the reuptake of 5-HT produces 5-HT7R-dependent effects and acts synergistically with a2AR activation [62]. The blockade of 5-HT3Rs also indicates that 5-HT exerts its effects on DNIC through these receptors. However, the effects of fluoxetine have been recently shown to be mediated through 5-HT7Rs but not 5-HT3Rs [136]. Thus, the effects of the serotonergic component of dual drugs might be exerted through 5-HT7Rs and also likely via 5-HT3Rs. The effects triggered by 5-HT3R activation, which are opposite to those of 5-HT7Rs, might possibly be balanced towards the beneficial effects of 5-HT7Rs, which are facilitated by the synergy with a2ARs through mechanisms that are still unknown. Supporting this, clinical studies suggest that duloxetine, a dual reuptake inhibitor, is an effective approach for managing chronic pain.

## Data Availability

No new data were created or analyzed in this study. Data sharing is not applicable to this article.
